# Technologies in Intensive Care Therapy and the Obstacles in Nursing Practice: Systematic Review

**DOI:** 10.1111/wvn.70124

**Published:** 2026-02-18

**Authors:** Antonio Henrique Braga Martins de Aguiar, Patrícia Ribeiro Azevedo, Wildilene Leite Carvalho, Rodrigo Alves Marques, Cibele Silva Lima, Maria Almira Bulcão Loureiro, Rosilda Silva Dias, Andrea Cristina Oliveira Silva

**Affiliations:** ^1^ Federal University of Maranhão (UFMA) São Luís Maranhão Brazil; ^2^ School of Nursing Federal University of Maranhão (UFMA) São Luís Maranhão Brazil; ^3^ University Hospital of the Federal University of Maranhão (HUFMA‐EBSERH) São Luís Maranhão Brazil; ^4^ Graduate Program in Nursing (PPGENF) UFMA São Luís Maranhão Brazil; ^5^ Graduate Program in Nursing and at the School of Nursing UFMA São Luís Maranhão Brazil

**Keywords:** biomedical technology, critical care, intensive care units, nursing, systematic review

## Abstract

**Aim:**

To identify obstacles faced by nurses when using health technologies in Intensive Care Units (ICUs).

**Design:**

Systematic review following PRISMA and registered in PROSPERO.

**Methods:**

Six databases were searched. Two reviewers independently screened studies and appraised methodological quality using the Joanna Briggs Institute tool. Data were synthesized narratively.

**Results:**

Eight studies met eligibility criteria. Barriers clustered around limited training and technical competence, shorter professional experience, increased workload with multiple devices, organizational culture, and reduced direct patient contact, which may undermine patient‐centered care. Heterogeneity of study designs precluded meta‐analysis.

**Conclusions:**

Obstacles to technology use in ICUs arise from individual and organizational factors. Addressing these barriers requires structured education, mentoring for novice nurses, workload management, and supportive policies that integrate technology without displacing bedside care.

**Linking Evidence to Action:**

Nursing leaders and educators should implement ongoing, ICU‐specific technology training and mentoring. Managers and policymakers must ensure adequate staffing and promote Health Technology Assessment to align device implementation with clinical needs, safeguarding patient safety and the human dimensions of care.

## 1 Introduction

The incorporation of health technologies is essential to improving efficiency and safety in clinical care. These tools and techniques, when integrated into nursing practice, can enhance quality and reduce errors (Tanabe and Moreira 2021).

According to Nietsche (1999), the integration of such tools represents the materialization of procedures derived from understanding and utilizing a set of information. Furthermore, technologies in Nursing are categorized as follows: Administrative Technologies, Planning Technologies, Care Technologies, Process Communication Technologies, Patient Situation Analysis Technologies, and Educational Technologies. Therefore, understanding and mastering these tools in Nursing is fundamental.

Despite improvements in nursing practice processes through technological incorporation, obstacles persist in this context. Factors such as professional training rates, availability of adequate infrastructure, and continuous education can positively impact strategies for implementing a technological care environment (Machado et al. 2020).

In this review, *hard technologies* denote material, device‐based resources applied in intensive care, such as monitors, ventilators, infusion pumps, and dialysis machines that require specific competencies and can reorganize nursing workflows. *Social representations* are shared, collectively constructed forms of knowledge within professional groups that frame how technologies are perceived, valued, and incorporated into everyday nursing practice. These representations influence attitudes, learning needs, and the adoption of technology at the bedside.

In this sense, nursing practice in intensive care demands precise interpretation of human responses, appropriate selection of interventions, and continuous evaluation of outcomes. The ICU is a complex and dynamic environment that requires professionals with advanced skills, theoretical‐practical integration, keen perception, and assertive decision‐making (Linn et al. 2019). In light of these demands and the increasing integration of technologies in care practices, this review aims to identify the obstacles in the nursing practice process concerning health technology in Intensive Care Units.

## 2 Method

This systematic review was developed following the PRISMA protocol (Page et al. 2021). The review protocol was registered in the International Prospective Register of Systematic Reviews (PROSPERO). The guiding question was formulated using the PICO strategy, where P: population; I: intervention; C: comparison; O: outcome. Thus, we defined: P—ICU nursing teams; I—use of technologies; C—nursing teams not using technological resources; O—obstacles in the nursing practice process related to the incorporation of technologies. The guiding question was: What are the obstacles in the nursing practice process when using technologies in Intensive Care Units?

Inclusion criteria included primary studies with cross‐sectional, observational (cohort or case report), and non‐randomized clinical trial designs, covering all age groups and genders. Excluded were literature reviews, letters, editorials, guidelines, randomized clinical trials, and gray literature. The selected languages were English, Portuguese, and Spanish, with no restrictions on publication year. Randomized clinical trials were excluded because this review aimed to synthesize observational and cross‐sectional evidence that reflects real‐world nursing practices in ICUs. Furthermore, no RCTs specifically addressing nursing‐related technological obstacles were identified during the search process.

Searches were conducted in June 2023 in MEDLINE (PubMed), Embase, Scielo, LILACS, Web of Science, and Science Direct databases. For literature reviews, manual searches of references were performed to recover potentially relevant studies. Descriptors were pre‐selected through the Health Sciences Descriptors (DeCS) and Medical Subject Headings (MeSH) Terms: “Biomedical Technology,” “Nursing,” and “Intensive Care Units,” combined with their synonyms using Boolean operators AND and OR adapted for each database. Search strategies and the number of retrieved studies are presented in Table 1.

**TABLE 1:** Search strategies in databases.

Databases	Strategy search
MEDLINE (PubMed)	(“nursing” OR “nurse”) AND (“intensive care unit” OR “intensive therapy”) AND (“biomedical technology” OR “technology”)
Scielo	(*tecnologia biomédica) OR (tecnologias em saúde) AND (enfermagem) OR (cuidados de enfermagem) AND (unidades de terapia intensiva) OR (cuidados críticos)
EMBASE	((‘biomedical technology’ AND ‘nursing’ OR ‘nursing care’) AND ‘intensive care’ OR ‘intensive care nursing’)
LILACS	Tecnologia biomédica OR Tecnologias em Saúde AND Enfermagem OR Cuidados de enfermagem AND Unidades de Terapia Intensiva OR Cuidados críticos
Web of Science	Tecnologia biomédica OR Tecnologias em saúde AND Enfermagem OR Cuidados de enfermagem AND Unidades de terapia intensiva OR Cuidados críticos
Science Direct	“biomedical technology” AND “nursing” OR “nursing care” AND “intensive care units” OR “critical care”

In the first selection phase, two authors independently searched articles in the databases, selected potential studies by titles, and exported them to the reference manager Mendeley version 2.61.0. In the second phase, selected articles were transferred to Rayyan software for full abstract reading. In all phases, selection was independently and blindly conducted, with full‐text reading of articles, and a third author resolved inclusion divergences.

Data extraction was conducted using a proprietary form with information on publication year, country, authorship, objectives, methodology, results, and conclusions. Methodological quality was independently assessed by two authors using the Joanna Briggs Institute (JBI) tool; methodological quality was independently assessed by two reviewers using the JBI tool, which comprises nine items: (1) sampling frame appropriateness; (2) participant selection; (3) sample size; (4) detailed description of participants; (5) adequacy of data analysis coverage; (6) valid methods for condition identification; (7) standard and reliable measurement; (8) appropriate statistical analysis; and (9) response rate and its management.

Ethical approval was not required for this study because it is a systematic review that exclusively used secondary data extracted from previously published articles available in indexed databases. No human participants or animals were directly involved.

All ethical principles related to authorship, citation, and acknowledgment of intellectual property were strictly observed throughout the research and manuscript preparation. Each included source was accurately referenced, ensuring transparency, academic integrity, and respect for the original contributions of the cited authors.

This study adheres to the ethical standards of scientific publication established by the Committee on Publication Ethics (COPE) and follows the integrity guidelines recommended for systematic reviews.

## 3 Results

**FIGURE 1:**
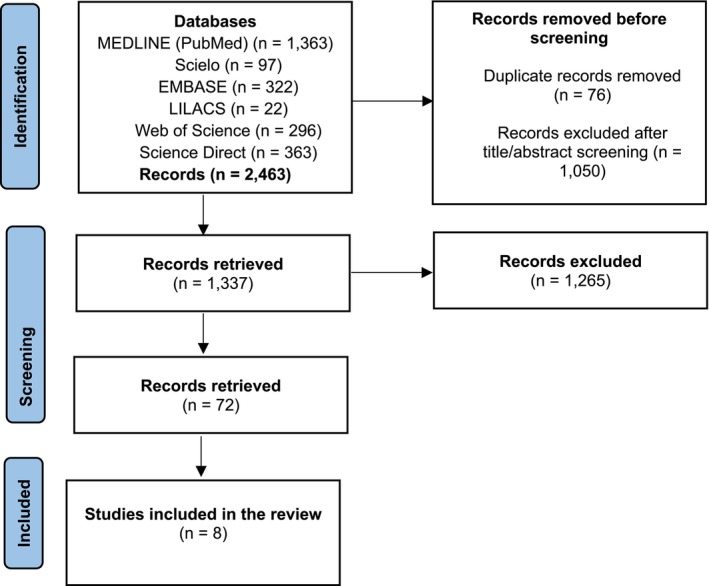
PRISMA flowchart of studies identified through databases (Page et al. 2021).

A total of 2463 articles were identified in the databases. After initial screening, removing duplicates, and excluding titles not meeting the objective, 1337 records remained. Subsequently, after a second screening based on inclusion and exclusion criteria and full‐title and abstract reading, eight articles were selected for data extraction and qualitative synthesis. The identification of studies in databases is illustrated in the PRISMA flowchart (Figure 1).

### 3.1 Study Characteristics

Most studies had a cross‐sectional design conducted via interviews (Nietsche 1999; Haakma et al. 2022; Silva et al. 2014, 2019; Ribeiro et al. 2016; Da Silva and Ferreira 2011) and in adult ICUs (Tunlind et al. 2015; Bagherian et al. 2017; Haakma et al. 2022; Silva et al. 2014, 2019; Ribeiro et al. 2016; Da Silva and Ferreira 2011). Regarding publication year, two were published in the last 5 years (Haakma et al. 2022; Silva et al. 2019), five in the last 10 years (Holden et al. 2016; Tunlind et al. 2015; Bagherian et al. 2017; Silva et al. 2014; Ribeiro et al. 2016) and one over a decade ago (Da Silva and Ferreira 2011). Four studies were published in Brazil (Silva et al. 2014, 2019; Ribeiro et al. 2016; Da Silva and Ferreira 2011), one in the Netherlands (Haakma et al. 2022), one in Iran (Bagherian et al. 2017), one in Sweden (Tunlind et al. 2015) and one in the United States (Holden et al. 2016). Table 2 summarizes the key characteristics of the included studies.

**TABLE 2:** Characterization of the studies included in the systematic review.

**Articles**	[1] Holden et al. (2016)	[2] Tunlind et al. (2015)	[3] Bagherian et al. (2017)
**Year**	2016	2014	2016
**Country**	United States	Sweden	Iran
**Author**	Holden et al.	Tunlind et al.	Bagherian et al.
**Aim**	To analyze the perception of pediatric ICU nurses regarding the acceptance and use of a customizable interactive monitor.	To describe the experiences of intensive care nurses in providing nursing care in a high‐technology healthcare environment.	To verify the association between the attitudes of intensive care nurses toward technology influences and their care attributes.
**Methods**	Cross‐sectional study with 167 nurses. A 28‐item instrument was used to assess ease of use, satisfaction, utility in family involvement, social influence, and training. Analyses were conducted using Cronbach's coefficient and multiple regression.	Cross‐sectional study with 8 nurses through interviews. Analyses were conducted using content analysis.	Cross‐sectional study with nurses from critical care units in teaching hospitals of Kerman University of Medical Sciences. A care provision attributes questionnaire was used to examine care attributes.
**Results**	Participants had moderate or higher scores for ease of use of the monitor and low scores for utility.	Care required more effort from the team when using multiple devices. Mobilization and hygiene were avoided when patients used tracheostomies, humidifiers, and dialysis tubes. The dialysis machine was considered the greatest obstacle in the time spent providing care.	Attribute scores decreased with age and experience, and commitment to care was more significant among older and more experienced nurses. Younger and less experienced nurses presented negative views about the technological impact on care.
**Conclusion**	The article strongly refutes the notion that the implementation of technologies in healthcare results in actual use.	Care was influenced by the use of devices on the patient.	Most negative attitudes regarding the technological influence in nursing stem from a lack of knowledge.

**Articles**	[4] Haakma et al. (2022)	[5] Silva et al. (2014)	[6] Silva et al. (2019)
**Year**	2022	2014	2019
**Local**	Netherlands	Brazil	Brazil
**Author**	Haakma et al.	Da Silva and Ferreira	Silva et al.
**Objective**	To explore the experiences of ICU nurses with the process of implementation and applicability of the Post‐ICU Diary.	To analyze the social representations of nursing care practices in the context of technologies applied to hospitalized ICU patients.	To identify the nursing team's perception of hard technology in care within the ICU environment.
**Method**	Exploratory pilot study with a non‐random sample conducted through individual interviews with nurses. Carried out in three ICUs. Thematic analysis of the data was performed.	Field study, qualitative, with 21 nurses, applying the Theory of Social Representations. Data collection was conducted through individual interviews, and the analysis was based on thematic content.	Descriptive‐exploratory, qualitative study with 36 nursing professionals working in ICUs. Convenience sampling was used, with data collected through semi‐structured interviews, recorded, transcribed, and analyzed lexically using the Iramuteq software.
**Results**	The use of the diary was considered difficult by the interviewees. They reported that logging in was not easy, which created a barrier to its use.	A lack of technical‐scientific training among part of the team in handling equipment and interpreting signals from it may harm the patient's recovery.	Hard technology in the environment can negatively interfere with care, as it represents an additional element requiring the team's attention, leading to reduced contact time with the patient and impacting the humanization of care.
**Conclusion**	Nurses confirmed the added value of the digital post‐ICU diary. However, they faced barriers related to its use, including a lack of time, insufficient integration with their work processes, and difficulties in writing short messages.	The content of the Social Representations (SRs) revealed elements that both integrate and shape the perspective of action assumed by the professional. During the formation process of these SRs, the prominence of technology in the care of ICU patients was observed.	Technology, despite providing speed and quality in care, paradoxically led to a distancing of the nursing team from the patient's bedside.

**Articles**	[7] Ribeiro et al. (2016)	[8] Da Silva and Ferreira (2011)
**Year**	2016	2011
**Local**	Brazil	Brazil
**Author**	Ribeiro et al.	Silva et al.
**Objective**	To identify the occurrence of errors in the use of equipment by nurses working in intensive care and analyze them through the lens of James Reason's human error theory.	To identify the social representations of nurses regarding technology applied in intensive care and relate them to their approaches to patient care.
**Method**	Qualitative field study with 8 nurses in the ICU of a hospital in Rio de Janeiro. Observations and interviews were conducted. Content analysis and description of the observed scenes were used for the interviews.	Qualitative study using the theoretical framework of social representations. Interviews were conducted with 24 nurses, accompanied by systematic observation and thematic content analysis.
**Results**	Memory and attention lapses were identified in the handling of infusion pumps, as well as planning failures during the programming of monitors.	Technology is unfamiliar to novice nurses, especially when combined with an unknown work environment, creating challenges in providing care. A lack of understanding of the technology distances nurses from the patient.
**Conclusion**	The authors propose the creation of a daily equipment checklist, including verifications throughout the work process for the programming of infusion pumps and monitors, to reduce failures and memory lapses.	The social representations of technology among nurses are shaped by specialized knowledge and practical experience. Technological and care‐related expertise, acquired through experience, influences the perceptions of both novice and veteran nurses regarding technology.

Methodological quality assessment of the studies was conducted using the JBI tool and is exemplified in Table 3.

**TABLE 3:** Methodological quality assessment of the studies according to the Joanna Briggs Institute tool.

Author	Item 1	Item 2	Item 3	Item 4	Item 5	Item 6	Item 7	Item 8	Item 9	Score
Holden et al.	Yes	Yes	Yes	No	Yes	Yes	Yes	Yes	Yes	Good
Tunlind et al.	No	No	No	No	Yes	No	Yes	No	No	Moderate Regular
Bagherian et al.	Yes	Yes	Yes	No	Yes	No	Yes	No	Yes	Good
Haakma et al.	No	No	No	Yes	Yes	Yes	Yes	Yes	Yes	Good
Da Silva and Ferreira	Yes	Yes	Yes	Yes	Yes	No	Yes	No	Yes	Intermediate
Silva et al.	Yes	Yes	Yes	Yes	Yes	Yes	Yes	Yes	Yes	Good
Ribeiro et al.	No	No	No	Yes	Yes	No	Yes	No	Yes	Regular
Silva et al.	Yes	Yes	Yes	Yes	Yes	No	Yes	No	Yes	Intermediate

## 4 Discussion

The results of this review revealed that the activities of nurses working in intensive care units are strongly influenced by technologies and innovative dynamics. Technologies play a significant and constructive role in their professional functions; however, inherent obstacles and challenges to their adoption and implementation are equally evidente (Lima et al. 2019).

It is important to emphasize the association between the use of technologies in care and the safety of the nursing team regarding their utilization, enabling efficient, safe, and effective care (Nascimento 2021).

Regarding the publication location, four studies were published in Brazil. From this perspective, the incorporation of technologies in healthcare services has been significantly implemented in recent decades and was initially recognized in developed countries such as Sweden and the Netherlands, as observed in two of the studies included in this review (Novaes and Soarez 2020).

The four Brazilian studies highlighted barriers to using these resources in intensive care. In this regard, it is noteworthy that since 2010, Health Technology Assessment (HTA) Centers have been instituted nationally in Teaching Hospitals across all regions, aiming to introduce the culture of HTA in hospital units. However, this strategy is still not a reality, despite favorable experiences already reported (Fernando and Malik 2019).

Two studies (Silva et al. 2019; Da Silva and Ferreira 2011) demonstrated that the incorporation of technologies in intensive care could lead to distancing by nurses with little professional experience. It also demands more time from the team, consequently reducing the time spent with patients. These factors, combined with the work environment, patients' clinical conditions, responsibilities, and qualifications and competencies of professionals, play a fundamental role in the panorama of nursing practice associated with the use of these resources (Almenyan et al. 2021).

Analyzing the objectives of the studies revealed a central focus on identifying obstacles, experiences, and attitudes of the nursing team concerning technology in the ICU context. Together, these objectives reflect a broad approach with a unique perspective on how technology is being incorporated, perceived, and its influences on intensive care.

All authors (Holden et al. 2016; Tunlind et al. 2015; Bagherian et al. 2017; Haakma et al. 2022; Silva et al. 2019; Ribeiro et al. 2016; Da Silva and Ferreira 2011) highlighted the relevance of using technology in a critical care environment. By incorporating technological advances, it is possible to observe benefits that resonate with both patients and multidisciplinary team professionals. However, it is crucial to recognize the challenges that may directly influence care quality and lead to adverse events compromising patient safety (Siman et al. 2019). Overcoming these obstacles is vital not only for continuous care improvement but also for enhancing operational efficiency and collaboration within the healthcare team (Pissaia et al. 2018).

The study by Silva et al.  (2014) (Lima et al. 2019) suggested that how professionals integrate these technological resources into their daily practice can exert positive or negative influences in the care context. This scenario raises reflections on the strategies adopted by professionals to interact with technologies, considering not only the positive influence but also the emotional impact and interpersonal relationship in patient care (Lima et al. 2019). Furthermore, the positive relationship between care attributes and technology influence highlights that nurses more committed to patient attention tend to see technology as an ally. However, identifying negative attitudes among novice nurses reveals the need for more comprehensive education about the role and impact of technology in care. Perissé et al. (2019) highlighted the challenges faced by younger nurses in dealing with technology and emphasized the importance of guidance programs, specialized training, and familiarization with technological language to ensure effective patient care.

Another aspect presented in this research focuses on the influence of technology on providing patient‐centered care. Medeiros et al. (2021) emphasized the importance of integrality in care processes, highlighting its influence on work dynamics in healthcare. Additionally, their study underscores the impact of this measure on directing comprehensive actions regarding the multifaceted dimensions of the individual. From this perspective, three studies (Tunlind et al. 2015; Silva et al. 2014, 2019) highlighted the need to balance technology with maintaining the ethical principles guiding nursing practice. Although technology is a valuable resource, its conscious and effective care use requires solid professional training that comprehends both the advantages and limitations of these advances (Júnior et al. 2018).

Regarding the technology adoption by the team, some studies (Holden et al. 2016; Haakma et al. 2022; Ribeiro et al. 2016) addressed the discrepancy between perceived ease of use and actual utility for patient care. Perniciotti et al. (2020) identified factors impacting nursing care, such as workload, extensive working hours, stress, exhaustion, and even absenteeism. This finding corroborates the selected studies, revealing that the ability to operate technology does not guarantee its effective care in improving care. Emphasis on low utility scores, particularly for patient care, indicates a disconnect between technology design and actual clinical needs.

The decision to use the JBI methodological quality assessment tool was based on its recommendation and frequent use for evaluating studies with a cross‐sectional design, like the ones included in this review. This tool is grounded in evidence‐based practice, focusing on the best available information and adaptable to diverse healthcare contexts to present evidence on a specific subject (Santos et al. 2018).

The selected articles, in general, reinforce the complexity of the obstacles and challenges faced by nursing professionals when using technological resources in intensive care. Each article exposes the perspective of its authors, converging on the need to balance technology use and patient‐centered care, alongside a continuous and adaptive approach to overcoming these challenges.

This study can support the formulation and implementation of strategies related to permanent education, resulting in effective care and effective care. Additionally, a significant point that supported this study's development was the search strategy stages, descriptor selection, and database selection, which were accompanied and reviewed by a librarian experienced in health research, allowing for its execution per the methodological rigor required for systematic reviews.

The findings have direct implications across nursing roles. Bedside nurses require ongoing, ICU‐specific training to operate devices safely while preserving patient‐centered care. Nurse educators should embed technological competence and humanization strategies into curricula. Managers must ensure supportive environments with adequate staffing and workload management. Policymakers should prioritize Health Technology Assessment and sustained professional development to align technology adoption with clinical needs.

To translate the evidence into practice, we synthesized the key findings into actionable recommendations, highlighting priority areas where nurses, educators, managers, and policymakers can intervene to overcome technological barriers and strengthen safe, patient‐centered care (Table 4).

**TABLE 4:** Linking evidence to action: practical implications for intensive care nursing.

Evidence from review	Recommended action
Limited technical‐scientific knowledge among nurses	Implement ongoing education programs focused on technology use in critical care
Limited professional experience increases barriers	Develop mentorship and preceptorship models to support novice nurses in ICUs
Equipment use creates distance from patient care	Encourage balanced protocols that prioritize patient‐centered care while using technologies
Organizational culture influences technology adoption	Promote institutional policies that integrate HTA and continuous technology assessment
Nurses experience stress and overload with multiple devices	Provide adequate staffing, workload management, and ergonomic interventions

As limitations for this study's development, primary articles presented diverse results, making it impossible to perform a meta‐analysis. It is suggested that studies focusing on this theme be developed to generate results and evidence significantly impacting nursing care for critically ill patients.

## 5 Conclusion

Nurses' use of technology in ICUs is constrained by gaps in technical training, limited experience, competing workload demands, and organizational factors that may inadvertently reduce direct patient contact. Targeted education, mentoring, staffing strategies, and HTA‐informed policies are pivotal to integrate technology while safeguarding patient‐centered care.

## Funding

The authors have nothing to report.

## Conflicts of Interest

The authors declare no conflicts of interest.

## Data Availability

The authors have nothing to report.
